# Intoxication by Asphyxiating Gas and Its Treatment

**Published:** 1918

**Authors:** 


***Intoxication by Asphyxiating Gas and Its Treatment.*** By ARMAND COLARD and PAUL SPEHL. Trans. and Abs. from the *Archives Médicales Belges,* July, 1917.

The authors make the following classification according to the respiratory phenomena:

A. Very acute and pronounced asphyctic syndrome, with deep cyanosis, blood vomiting, and early death.

B. Acute asphyctic syndrome following only slight symptoms of intoxication.

C. Syndrome of caustic tracheo-bronchitis.

The cases considered in the article belong to groups B and C.

*Group B.* — As soon as the gas has been inhaled there is an acute irritation of the respiratory and upper digestive tracts, which may even cause nausea. The symptoms may subside for a short period, but a few hours later dyspnea increases gradually. When admitted to the hospital, these patients show all the symptoms of acute asphyxia.

In all the patients observed, except one, the respiration was shallow and rapid. The most serious case, however, had a slow and very painful respiration with inspiratory and expiratory dyspnea.

As a rule the pulse is even and regular, but rapid.

Blood tests indicate a slight deficiency of red corpuscles. The viscosity is increased and the blood of the median cephalic vein is black and thick.

Auscultation of râles in all of the cases proved that the irritating action of the gas affected the smallest bronchial ramifications.

Tracheo-bronchitis may last several weeks with a persistent retrosternal pain and great sensitiveness of the trachea on palpation.

In all the cases at the end of two or three days the sputum was of a dark yellowish color. Later it became muco-purulent and sometimes it contained blood.

*Group C.* — The symptoms of the first few hours were like those of group B. Instead of the asphyxiating complications observed after the recrudescence in group B, patients of group C continue with tracheo-bronchitis. When the asphyxia has disappeared, the two groups again have similar symptoms. In group C the râles may be heard for about six days, although they sometimes disappear in two. The temperature generally falls on the second or third day, and then the pulse returns to normal. Even if every serious symptom has disappeared, gas cases must be watched carefully, especially during the second week.

*Complications.* Broncho-pneumonia is much to be feared. This may cause gangrene or tuberculosis. Bleeding and prolonged inhalation of oxygen seem to have been the most efficacious form of treatment. Weakness of the pulse is not, in cases of severe intoxication, a contra-indication of bleeding. From 300 to 500 cc. of blood should be removed as early as possible.

The oxygen should be administered under constant pressure and continuously. The patient should be kept in an atmosphere of pure oxygen until all alarming phenomena have disappeared, even if this requires a continuation of the oxygen supply for several days.

The apparatus used enables several patients to be supplied from the same tank at the same time.

Good results have also been obtained by the use of digitaline.

